# Adherence to 24-Hour Movement Guidelines Among Chinese Older Adults: Prevalence, Correlates, and Associations With Physical and Mental Health Outcomes

**DOI:** 10.2196/46072

**Published:** 2024-06-13

**Authors:** Wei Liang, Yanping Wang, Qian Huang, Borui Shang, Ning Su, Lin Zhou, Ryan E Rhodes, Julien Steven Baker, Yanping Duan

**Affiliations:** 1 School of Physical Education Shenzhen University Shenzhen China; 2 Department of Sport, Physical Education and Health Hong Kong Baptist University Hong Kong China (Hong Kong); 3 Fitness and Health Lab Hubei Institute of Sport Science Wuhan China; 4 Department of Social Sciences Hebei Sports University Shijiazhuang China; 5 School of Physical Education Hebei Normal University Shijiazhuang China; 6 School of Exercise Science, Physical and Health Education University of Victoria Victoria, BC Canada

**Keywords:** physical activity, sedentary behavior, sleep, cardiometabolic indicators, physical fitness, mental health, post–COVID-19 era, older adults, COVID-19, systolic blood pressure, diastolic blood pressure, depression, loneliness

## Abstract

**Background:**

It is known that 24-hour movement behaviors, including physical activity (PA), sedentary behavior (SB), and sleep, are crucial components affecting older adults’ health. Canadian 24-hour movement guidelines for older adults were launched in 2020, emphasizing the combined role of these 3 movement behaviors in promoting older adults’ health. However, research on the prevalence and correlates of guideline adherence and its associations with health-related outcomes is limited, especially among Chinese older adults.

**Objective:**

This study aimed to investigate the prevalence and correlates of meeting 24-hour movement guidelines among Chinese older adults. Furthermore, this study aimed to examine the associations of guideline adherence with older adults’ physical and mental health outcomes.

**Methods:**

Using a stratified cluster random sampling approach, a total of 4562 older adults (mean age 67.68 years, SD 5.03 years; female proportion: 2544/4562, 55.8%) were recruited from the latest provincial health surveillance of Hubei China from July 25 to November 19, 2020. Measures included demographics, movement behaviors (PA, SB, and sleep), BMI, waist circumference, waist-hip ratio (WHR), percentage body fat (PBF), systolic and diastolic blood pressure, physical fitness, depressive symptoms, and loneliness. Generalized linear mixed models were employed to examine the associations between variables using SPSS 28.0 (IBM Corp).

**Results:**

Only 1.8% (83/4562) of participants met all 3 movement guidelines, while 32.1% (1466/4562), 3.4% (155/4562), and 66.4% (3031/4562) met the individual behavioral guidelines for PA, SB, and sleep, respectively. Participants who were older, were female, and lived in municipalities with lower economic levels were less likely to meet all 3 movement guidelines. Adhering to individual or combined movement guidelines was associated with greater physical fitness and lower values of BMI, waist circumference, WHR, PBF, depressive symptoms, and loneliness, with the exception of the relationship of SB+sleep guidelines with loneliness. Furthermore, only meeting SB guidelines or meeting both PA and SB guidelines was associated with lower systolic blood pressure.

**Conclusions:**

This is the first study to investigate adherence to 24-hour movement guidelines among Chinese older adults with regard to prevalence, correlates, and associations with physical and mental health outcomes. The findings emphasize the urgent need for promoting healthy movement behaviors among Chinese older adults. Future interventions to improve older adults’ physical and mental health should involve enhancing their overall movement behaviors and should consider demographic differences.

## Introduction

The number of older adults (≥60 years) worldwide was estimated to be 1 billion in 2019, and this figure is expected to double by 2050, accounting for around 22% of the global population [1]. This demographic shift has been occurring at an unprecedented pace and may accelerate in the coming decades, and it will bring considerable challenges to worldwide societies, especially in developing countries [1,2]. As a vulnerable group, older adults have shown low levels of physical fitness and high levels of morbidity and mortality of infectious respiratory diseases, cardiometabolic diseases, and mental disorders (eg, late-life depression), which have worsened because of the outbreak and continuation of the COVID-19 pandemic [3-5]. The daily routines of older adults have been substantially altered [6]. They have been challenged by requirements to increase their time living at home, limits to physical and social connections with other family members and friends, temporary decreases or cessation of employment and recreational activities, loneliness, and fear of illness and death for themselves and others [3,6-8]. Therefore, promoting physical and mental health among older adults during the pandemic and beyond to achieve healthy aging is a public health and socioeconomic imperative globally [7].

It has been shown that 24-hour movement behaviors, including physical activity (PA), sedentary behavior (SB), and sleep, have prominent impacts on a wide range of physical and mental health outcomes among older adults [9-11]. For example, regular engagement in PA has been shown to be reliably associated with better health-related outcomes, such as BMI [12,13], percentage body fat (PBF) [12,13], waist circumference [14,15], waist-hip ratio (WHR) [15,16], systolic and diastolic blood pressures (SBP and DBP, respectively) [17], physical fitness [18,19], and depression and loneliness [20-22]. Similarly, adequate sleep duration has been shown to be associated with greater cardiorespiratory fitness [23,24] and decreased risks of metabolic diseases [25] and mental disorders [26] among older adults. In contrast, prolonged sedentary time has been shown to be a modifiable risk factor that negatively affects the physical and mental health of older adults [19,27-29].

Historically, most studies have focused only on the effect of one of these specific movement behaviors on health-related outcomes, whereas the interrelationship and joint effects of these movement behaviors were comparatively ignored [30,31]. In recent decades, an increasing collection of evidence has supported the combined effects on health among different populations, including preschool children [32], school children [33-36], adolescents [33,36], and adults and older adults [30,31]. Drawing on the theoretical underpinnings of time-use epidemiology and bolstered by recent empirical findings, the Canadian 24-hour Movement Guidelines for adults aged 18-64 years and those aged 65 years or older were established in 2020 [31]. These guidelines advocate for adults aged 18 years or older to engage in a minimum of 150 minutes of moderate-to-vigorous physical activity (MVPA) cumulatively per week. Additionally, they recommend limiting SB to no more than 8 hours daily, with recreational screen time not exceeding 3 hours, and maintaining a sleep duration of 7-9 hours for adults aged 18-64 years and 7-8 hours for those aged 65 years or older. Furthermore, the guidelines emphasize the importance of regular sleep and wake-up times.

The launch of 24-hour movement guidelines for adults has inspired relevant research interest in the combination of 24-hour movement behaviors among adult populations [37-42]. For instance, a national-level surveillance (2007-2013) found that Canadian adults (aged 18-79 years) who adhered to all 3 movement guidelines had more favorable BMI, waist circumference, aerobic fitness, and cardiometabolic biomarker levels (eg, C-reactive protein and insulin levels) [38]. A recent cross-sectional study indicated an inverse association between meeting 24-hour movement guidelines and mental health outcomes (eg, depression) among Chinese caregivers of preschoolers (mean age 35.5 years, SD 4.9 years) during the COVID-19 pandemic [41]. However, there is a lack of evidence among Chinese older adults.

Previous studies have found that meeting 24-hour movement guidelines was correlated with a range of demographic factors, such as sex, education level, marital status, health condition, and economic status among adult populations [37,38,42]. However, all the above studies focused on young individuals and middle-aged adults, while to the best of our knowledge, there is a scarcity of recent evidence on the correlates of meeting 24-hour movement guidelines among older adults (aged ≥60 years).

Therefore, this study aimed to (1) investigate the prevalence of meeting 24-hour movement guidelines among Chinese older adults; (2) investigate the correlates of meeting 24-hour movement guidelines among Chinese older adults; and (3) examine the association of adherence to 24-hour movement guidelines with physical (ie, BMI, waist circumference, WHR, PBF, SBP, DBP, and physical fitness) and mental (ie, depression and loneliness) health outcomes among Chinese older adults.

## Methods

### Participants and Procedure

Participants were recruited from the latest provincial health surveillance of Hubei China (HSHC) [43]. The HSHC is an ongoing consecutive cross-sectional surveillance that collects various health indicators on a representative sample of Chinese residents living in the Hubei province of China every 5 years [43]. A self-weighted stratified cluster random sampling approach was applied in the HSHC, where participants were randomly selected from 17 municipalities of Hubei province, stratified by communities, towns (villages), and districts (counties) for each municipality. In this study, eligible participants were required to meet the inclusion criteria, including (1) age of 60-79 years, (2) adequate language skills (ie, reading and writing capabilities in Chinese), and (3) no restriction of physical mobility (eg, passed the Physical Activity Readiness Questionnaire [PAR-Q]). Participants excluded from this study were those outside the specified age range, those unable to read and comprehend Chinese, those failing the PAR-Q, and those diagnosed with cognitive or sleep disorders. A total of 32,080 participants were contacted, and 27,826 agreed to participate in the surveillance (86.7% response rate). After eligibility checks, 4953 eligible participants were invited to complete data collection.

Eligible participants were further invited to complete the study measures at a multi-function stadium, lasting approximately 30 minutes per person. To ensure assessment quality, for each municipality, the data collection was conducted by a trained health surveillance team, which consisted of 15-20 qualified assessors who passed a competency examination, according to consistent standard operating procedures. Data were collected from July 25, 2020 (3 months after the lockdown was withdrawn) to November 19, 2020.

### Ethical Considerations

This study followed the Declaration of the Helsinki World Medical Association [44] and the STROBE (Strengthening the Reporting of Observational Studies in Epidemiology) statement [45]. Ethics approval was obtained from the General Administration of Sport of China (CISS-2019-01-31) and Hubei Institute of Sport Science (HISS-2019-03-01). All participants who were interested in participating in the surveillance were asked to sign a written informed consent form before the study commencement.

### Measures

#### Adherence to 24-Hour Movement Guidelines

The Chinese version of the International Physical Activity Questionnaire long form (IPAQ-LC) was used to measure PA, SB, and sleep (interconsistency coefficient [ICC]=0.79-0.87) [46,47]. For PA, participants were asked to report the frequency (days) and duration (minutes) of 3 intensities of PA (ie, light PA, moderate PA, and vigorous PA) during the past 7 days. Weekly time of PA was calculated using the following formula: frequency×duration. For sedentary and sleep time, participants were asked to report the time (hours and minutes per day) they spent in these 2 behaviors on weekdays and weekends separately during the past week. Daily time of SB and sleep was calculated by dividing the weekly time of SB and sleep by 7 days. According to the Canadian 24-hour movement guidelines [31], participants were categorized as 0 (meeting none of the movement guidelines), 1 (meeting 1 of the movement guidelines), 2 (meeting 2 of the movement guidelines), and 3 (meeting all 3 movement guidelines).

#### Physical Health Outcomes

Physical health outcomes included objectively measured BMI, waist circumference, WHR, PBF, SBP, DBP, and physical fitness. Participants were informed to not participate in any vigorous PA 12 hours before the assessment. The assessments were conducted at indoor multi-function sport gymnasiums, with ambient temperature kept constant during the measurements for all participants.

Body weight and body height were measured using a portable stadiometer (GMCS-SGJ3; to the closest 0.05 kg) and a calibrated medical digital scale (GMCS-RCS3), which were further used to calculate BMI (kg/m^2^) [48]. Waist and hip circumference were measured using specific tape measures (GMCS-WD3; to the closest 0.1 cm). WHR was calculated as waist circumference (cm) divided by hip circumference (cm). PBF was measured using a portable bioelectrical impedance device (GMCS-TZL3). Participants were measured after either an overnight or 2-hour fast and were asked to remove their footwear and socks before stepping on to the measurement instrument. The whole assessment followed a standard procedure (eg, placing the feet on 4 pads, keeping the arms straight down, and not touching the inner thighs) guided by qualified assessors. SBP and DBP were measured after participants sat for 15 minutes, using digital instruments (GMCS-XY3).

Physical fitness was measured according to the standard protocol of the National Physical Fitness Surveillance of China [49,50]. The entire physical fitness assessment included 7 tests in this study: (1) vital capacity test using a spirometer (GMCS-FHL), (2) handgrip strength test using a mechanical dynamometer (GMCS-WCS3), (3) chair sit-and-reach test, (4) 30-second chair stand test, (5) 2-minute step test, (6) 1-leg standing with eyes closed balance test, and (7) choice reaction time test using a traditional test plate (GMCS-FYS). Each test was conducted twice, and the best performance of the 2 trials was recorded for analysis. The total physical fitness score was the sum of the weighted score of each test, ranging from 10 to 100, with a higher score indicating a greater physical fitness [49,50]. Prior to testing, all participants were fully familiarized with the measurement procedures.

#### Mental Health Outcomes

Depression was measured using the Chinese version of the Patient Health Questionnaire-9 (PHQ-9) [51,52]. Following the instruction question “how often were you bothered by the following problems over the past two weeks…,” participants were asked to give answers to 9 situations (eg, “little interest or pleasure in doing things”) on a 4-point Likert scale ranging from 0 (“not at all”) to 3 (“nearly every day”) (Cronbach α=.88). The total score of the 9 items was calculated (range 0-27), with a higher score indicating a more severe level of depressive symptoms.

Loneliness was measured using the Chinese version of the 10-item Emotional and Social Loneliness Scale (ESLS-10) [53,54]. Participants were asked to answer how often the 10 designated feelings occurred over the past year (eg, “I feel as if nobody really understands me”). Responses were indicated on a 5-point Likert scale ranging from 1 (“not at all”) to 5 (“very often”) (Cronbach α=.85). The total score of the 10 items was calculated (range 10-50), with a higher score reflecting a higher level of loneliness.

#### Covariates

Covariates were chosen in accordance with prior research [30,31,37] and included age, gender, place of residence (urban/rural), educational attainment, marital status, chronic diseases (eg, hypertension, cardiovascular diseases, stroke, osteoporosis, cancer, and type 2 diabetes), current smoking and alcohol consumption behaviors, and the economic status of municipalities (as determined by provincial gross domestic product [GDP] statistics) [55].

### Statistical Analysis

Data analyses were performed using SPSS 28.0 (IBM Corp). We excluded 248 cases owing to missing demographic details (73 cases lacking age and gender information), movement behaviors (73 cases), and physical and mental health outcomes (248 cases without data on BMI, PBF, physical fitness, and 2 mental health indicators) and 143 cases owing to invalid or abnormal values in movement behaviors (122 cases) and health-related outcomes (76 cases; eg, BMI and PBF), particularly those cases with skewness and kurtosis absolute values beyond ±1.5 and z-scores exceeding the ±3 SD threshold [56]. Thus, data from 4562 participants were retained for the final analysis. Based on a retrospective power estimate, the final sample size of 4562 was adequate to detect an effect size (Cohen *f*^2^) of 0.01, with an α of .05 and a statistical power (1-β) of 0.8, in the regression model [35,41]. Odds ratios (ORs) of 1.68, 3.47, and 6.71 were considered to indicate a small, medium, and large effect size, respectively [57]. A statistical significance level of *P*<.05 (2-tailed) with 95% CI not covering 0 was used for all analyses.

Mean, SD, and percentage (%) have been used to present the descriptive characteristics of the study sample. Considering that the 24-hour movement guidelines propose different requirements for those aged 60-64 years and those aged ≥65 years, demographic characteristics were also reported for each subgroup. For the main analyses, generalized linear mixed models were used to examine the correlates of meeting 24-hour movement guidelines and assess the associations of guideline adherence with physical and mental health outcomes.

Finally, sensitivity analyses were performed to examine the robustness of the study findings and alternative explanations. All the association analyses were reconducted with the exclusion of participants who were obese (BMI ≥28 kg/m^2^) or who had moderate to severe depressive symptoms (PHQ-9 score ≥10) as suggested by previous studies [58,59]. Additionally, e-values were computed for the primary outcomes to assess the potential bias from unmeasured and residual confounding factors [58-60]. E-values estimate the magnitude of unmeasured or residual confounders that would need to be present to nullify the established relationships between independent and dependent variables, despite adjustments for all identified covariates [58-60]. E-values exceeding 1 suggest that only confounders of significant magnitude could challenge the observed relationships, including a greater robustness of these results against the influence of unmeasured confounders [58-60].

## Results

### Sample Characteristics

**Figure 1:**
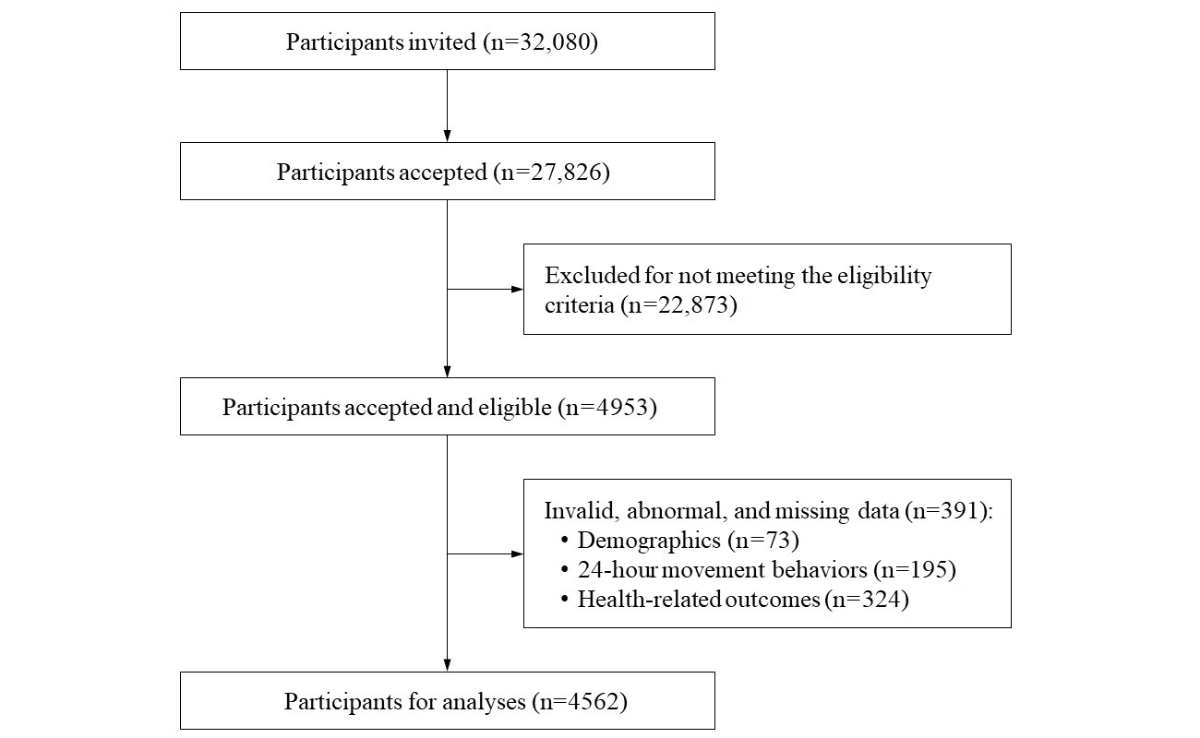
STROBE (Strengthening the Reporting of Observational Studies in Epidemiology) diagram of the study process.

Of 4953 participants, 4562 (retention rate of 92.1%) were included in the final data analysis (Figure 1). In addition, 69.2% (3157/4562) of participants were aged 65 years or older, and the mean age of the total sample was 67.68 (SD 5.03) years. Moreover, 55.8% (2544/4562) of participants were female and 53.0% (2417/4562) were living in urban areas. Participants with an education level of secondary school accounted for the largest proportion (2115/4562, 46.4%), while only 12.2% (555/4562) of participants had an education level of college or above. Furthermore, 86.9% (3964/4562) of participants were married, and 46.8% (2134/4562) reported a history of chronic diseases. Details of the study sample are presented in Table 1.

**Table 1:** Descriptive characteristics of the study sample.

Variable		Total (N=4562)	Age 60-64 years (n=1405)	Age ≥65 years (n=3157)
Age (years), mean (SD)		67.68 (5.03)	62.12 (1.36)	70.15 (4.00)
**Gender, n (%)**				
	Male	2018 (44.2)	619 (44.1)	1399 (44.3)
	Female	2544 (55.8)	786 (55.9)	1758 (55.7)
**Residence, n (%)**				
	Urban	2417 (53.0)	782 (55.7)	1635 (51.8)
	Countryside	2145 (47.0)	623 (44.3)	1522 (48.2)
**Education, n (%)**				
	Primary school or below	1892 (41.5)	456 (32.5)	1436 (45.5)
	Secondary school	2115 (46.4)	753 (53.5)	1362 (43.1)
	College or above	555 (12.2)	196 (14.0)	359 (11.4)
**Marital status, n (%)**				
	Married	3964 (86.9)	1277 (90.9)	2687 (85.1)
	Single/divorced/widowed	598 (13.1)	128 (9.1)	470 (14.9)
**Chronic disease, n (%)**				
	No	2428 (53.2)	836 (59.5)	1592 (50.4)
	Yes	2134 (46.8)	569 (40.5)	1565 (49.6)
**Smoking, n (%)**				
	Not currently	648 (14.2)	235 (16.7)	413 (13.1)
	Yes, but not everyday	184 (4.0)	65 (4.6)	119 (3.8)
	Yes, almost everyday	3730 (81.8)	1105 (78.6)	2625 (83.1)
**Alcohol, n (%)**				
	Never	3423 (75.0)	1030 (73.3)	2393 (75.8)
	Seldom	627 (13.7)	220 (15.7)	407 (12.9)
	Often	512 (11.2)	155 (11.0)	357 (11.3)
**Municipality economic status, n (%)**				
	≥9th in terms of provincial GDP^a^	2344 (51.4)	676 (48.1)	1668 (52.8)
	<9th in terms of provincial GDP	2218 (48.6)	729 (51.9)	1489 (47.2)
**Physical health outcomes, mean (SD)**				
	BMI (kg/m^2^)	24.36 (3.05)	23.91 (2.72)	24.55 (3.17)
	Waist circumference (cm)	86.46 (9.38)	84.72 (8.48)	87.24 (9.65)
	WHR^b^	0.92 (0.07)	0.91 (0.07)	0.93 (0.07)
	PBF^c^ (%)	27.92 (6.87)	27.14 (6.55)	28.27 (6.98)
	SBP^d^ (mmHg)	139.63 (18.52)	139.41 (18.49)	139.73 (18.55)
	DBP^e^ (mmHg)	82.67 (10.98)	82.81 (10.82)	82.61 (11.06)
	Physical fitness	59.50 (11.91)	60.10 (10.06)	59.24 (12.64)
**Mental health outcomes, mean (SD)**				
	Depression	2.59 (3.35)	1.99 (2.65)	2.86 (3.58)
	Loneliness	21.59 (7.91)	20.61 (7.24)	22.03 (8.15)

^a^GDP: gross domestic product.

^b^WHR: waist-hip ratio.

^c^PBF: percentage body fat.

^d^SBP: systolic blood pressure.

^e^DBP: diastolic blood pressure.

### Prevalence of Adherence to 24-Hour Movement Guidelines

**Figure 2:**
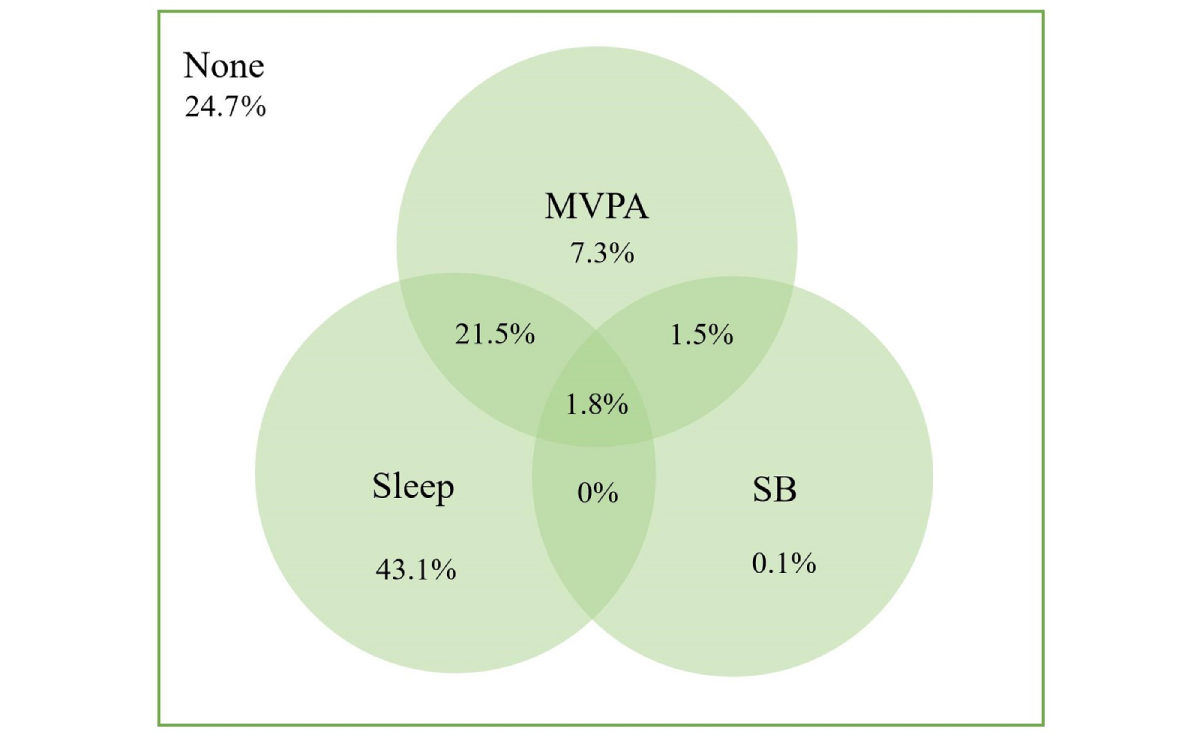
Adherence to 24-hour movement guidelines among the study participants. The value within each circle is added to the percentage of participants meeting each individual guideline (ie, 32.1% for MVPA, 3.4% for SB, and 66.4% for sleep). The overall nonoverlapped area of each circle refers to the percentage of participants meeting 1 of the 3 guidelines (ie, 7.3%+0.1%+43.1%=50.5%). The overall overlapped area of 2 circles refers to the percentage of participants meeting 2 movement guidelines (ie, 21.5%+1.5%+0%=23.0%). The overlapped area of 3 circles refers to the percentage of participants meeting all 3 movement guidelines (ie, 1.8%). The outside area of the circle refers to the percentage of participants meeting none (0) of the guidelines (ie, 24.7%). MVPA: moderate-to-vigorous physical activity; SB: sedentary behavior.

The rates of adhering to 0, 1, and 2 movement guidelines were 24.7% (1126/4562), 50.5% (2303/4562), and 23.0% (1050/4562), respectively. The proportion of participants meeting individual movement guidelines was 32.1% (1466/4562) for MVPA, 3.4% (155/4562) for SB, and 66.4% (3031/4562) for sleep. Prevalence rates of meeting a combination of 2 movement behaviors ranged from 1.8% (83/4562) to 23.3% (1063/4562), while only 1.8% (83/4562) of participants met all 3 movement guidelines (Figure 2).

### Correlates of Adherence to 24-Hour Movement Guidelines

Table 2 presents the correlates of meeting 24-hour movement guidelines in the study sample. Participants who were older and who were female had a comparatively poorer adherence to either individual movement guidelines or the combinations of 2 or 3 behaviors (all *P*<.001). The municipality economic status was positively associated with meeting all 3 movement guidelines. Participants living in urban areas were more likely to adhere to MVPA and sleep guidelines (all *P*<.001). A higher education level and no chronic diseases were associated with a higher adherence to MVPA, sleep, and MVPA+SB guidelines, while marital status was not associated with guideline adherence.

**Table 2:** Correlates of adherence to 24-hour movement guidelines in the study sample (N=4562).

Variable		MVPA^a^, OR^b^ (95% CI)	SB^c^, OR (95% CI)	Sleep, OR (95% CI)	MVPA+SB, OR (95% CI)	MVPA+sleep, OR (95% CI)	SB+sleep, OR (95% CI)	MVPA+SB+sleep, OR (95% CI)
Age		0.97 (0.95-0.98)^d^	0.93 (0.90-0.97)^d^	0.97 (0.96-0.98)^d^	0.94 (0.90-0.97)^d^	0.97 (0.96-0.99)^d^	0.94 (0.89-0.98)^e^	0.94 (0.89-0.98)^e^
**Gender**								
	Male (reference)	N/A^f^	N/A	N/A	N/A	N/A	N/A	N/A
	Female	0.55 (0.47-0.64)^d^	0.40 (0.27-0.60)^d^	0.55 (0.47-0.64)^d^	0.39 (0.26-0.59)^d^	0.56 (0.47-0.66)^d^	0.36 (0.21-0.61)^d^	0.35 (0.21-0.61)^d^
**Residence**								
	Urban (reference)	N/A	N/A	N/A	N/A	N/A	N/A	N/A
	Countryside	0.75 (0.66-0.86)^d^	0.83 (0.59-1.16)	0.78 (0.69-0.89)^d^	0.80 (0.57-1.13)	0.77 (0.66-0.89)^d^	0.82 (0.52-1.32)	0.83 (0.52-1.33)
**Education**								
	Primary school or below (reference)	N/A	N/A	N/A	N/A	N/A	N/A	N/A
	Secondary school	1.19 (1.03-1.37)^g^	0.93 (0.64-1.35)	1.06 (0.92-1.22)	0.96 (0.66-1.39)	1.21 (1.03-1.43)^g^	1.38 (0.80-2.39)	1.40 (0.81-2.41)
	College or above	1.41 (1.13-1.75)^e^	0.81 (0.47-1.40)	1.86 (1.46-2.38)^d^	0.82 (0.47-1.42)	1.54 (1.22-1.95)^d^	1.46 (0.71-3.01)	1.50 (0.73-3.07)
**Marital status**								
	Married (reference)	N/A	N/A	N/A	N/A	N/A	N/A	N/A
	Single/divorced/widowed	1.15 (0.95-1.39)	1.03 (0.61-1.74)	1.11 (0.92-1.33)	0.88 (0.50-1.55)	1.22 (0.99-1.51)	0.74 (0.31-1.72)	0.96 (0.45-2.03)
**Chronic diseases**								
	No (reference)	N/A	N/A	N/A	N/A	N/A	N/A	N/A
	Yes	0.87 (0.76-0.99)^g^	1.20 (0.86-1.67)	0.60 (0.53-0.69)^d^	1.20 (0.86-1.67)	0.85 (0.74-0.99)^g^	1.03 (0.66-1.62)	1.04 (0.66-1.63)
**Smoking**								
	Not currently (reference)	N/A	N/A	N/A	N/A	N/A	N/A	N/A
	Yes, but not everyday	0.63 (0.43-0.90)^g^	0.59 (0.19-1.30)	1.15 (0.79-1.67)	0.50 (0.19-1.29)	0.78 (0.52-1.16)	0.42 (0.10-1.83)	0.42 (0.10-1.85)
	Yes, almost everyday	1.13 (0.92-1.38)	0.90 (0.58-1.40)	1.05 (0.85-1.30)	0.89 (0.57-1.38)	1.18 (0.95-1.47)	0.94 (0.51-1.72)	0.95 (0.52-1.74)
**Alcohol**								
	Never (reference)	N/A	N/A	N/A	N/A	N/A	N/A	N/A
	Seldom	1.33 (1.09-1.63)^e^	1.12 (0.69-1.81)	0.93 (0.76-1.15)	1.13 (0.70-1.82)	1.24 (1.01-1.54)^g^	0.94 (0.48-1.81)	0.94 (0.49-1.81)
	Often	1.24 (0.99-1.54)	1.43 (0.89-2.28)	0.82 (0.66-1.04)	1.42 (0.89-2.28)	1.07 (0.84-1.35)	1.12 (0.89-2.13)	1.13 (0.59-2.15)
**Municipality economic status**								
	<9th in terms of GDP^h^ (reference)	N/A	N/A	N/A	N/A	N/A	N/A	N/A
	≥9th in terms of GDP	0.93 (0.82-1.07)	1.49 (1.06-2.09)^g^	0.83 (0.73-0.94)^e^	1.50 (1.06-2.10)^g^	0.94 (0.82-1.09)	1.67 (1.05-2.67)^g^	1.67 (1.04-2.66)^g^
R^2^		0.06	0.05	0.07	0.05	0.05	0.06	0.06

^a^MVPA: moderate-to-vigorous physical activity.

^b^OR: odds ratio.

^c^SB: sedentary behavior.

^d^*P*<.001.

^e^*P*<.01.

^f^N/A: not applicable.

^g^*P*<.05.

^h^GDP: gross domestic product.

### Associations of Adherence to 24-Hour Movement Guidelines With Physical and Mental Health Outcomes

The associations between adherence to movement guidelines (individual and combination) and health-related outcomes are outlined in Tables 3 and 4. Adhering to either MVPA or sleep guidelines was associated with favorable physical and mental health outcomes (all *P*<.001), except 2 blood pressure indicators (*P*=.08-.57). Similar results were observed for adherence to SB guidelines, except a significant correlation with SBP (*P*=.02).

Adhering to both MVPA and SB guidelines was associated with greater health-related outcomes (all *P*<.01), except DBP (*P*=.88). Adhering to MVPA+sleep or SB+sleep guidelines was associated with better performance regarding physical health (all *P*<.05), except 2 blood pressure indicators (*P*=.09-.78). Adhering to MVPA+sleep guidelines was associated with lower levels of both depressive symptoms and loneliness (both *P*<.001), while adhering to SB+sleep guidelines was not significantly associated with loneliness (*P*=.18).

Relative to not meeting any movement guidelines, adherence to 1, 2, or 3 movement guidelines was associated with lower values of BMI, waist circumference, WHR, and PBF, alongside higher levels of physical fitness among participants (all *P*<.001). However, this association did not extend to blood pressure (both SBP and DBP; *P*=.17-.89). For mental health outcomes, adherence to 1, 2, or 3 movement guidelines was significantly and inversely associated with both depressive symptoms and loneliness (all *P*<.001) compared with not meeting any guidelines (Table 4). Dose-response associations were identified between the number of adhered guidelines and health-related outcomes (all *P*<.01), except blood pressure (*P*=.18-.64).

**Table 3:** Associations of adherence to 24-hour movement guidelines with physical outcomes (N=4562).

Meeting movement guidelines^a,b^		BMI (kg/m^2^), *B* (95% CI)	WC^c^ (cm), *B* (95% CI)	WHR^d^, *B* (95% CI)	PBF^e^ (%), *B* (95% CI)	SBP^f^ (mmHg), *B* (95% CI)	DBP^g^ (mmHg), *B* (95% CI)	Physical fitness, *B* (95% CI)
**Meeting individual guidelines^h^**								
	At least MVPA^i^	–1.22 (–1.41 to –1.04)^j^	–3.84 (–4.40 to –3.27)^j^	–0.02 (–0.02 to –0.01)^j^	–2.44 (–2.75 to –2.12)^j^	–1.05 (–2.22 to 0.12)	–0.20 (–0.90 to 0.50)	2.99 (2.64 to 3.34)^j^
	At least SB^k^	–0.84 (–1.32 to 0.36)^j^	–2.80 (–4.25 to –1.35)^j^	–0.01 (–0.02 to –0.003)^l,m^	–2.37 (–3.19 to –1.56)^j^	–3.61 (–6.57 to –0.64)^l^	–0.01 (–1.77 to 1.76)	2.80 (1.90 to 3.71)^j^
	At least sleep	–0.51 (–0.70 to –0.32)^j,m^	–1.40 (–1.97 to –0.83)^j,m^	–0.01 (–0.01 to –0.005)^j^	–0.66 (–0.98 to –0.34)^j,m^	0.36 (–0.80 to 1.53)	–0.06 (–0.75 to 0.64)	1.65 (1.30 to 2.00)^j^
**Meeting specific guideline combinations^n^**								
	At least MVPA+SB	–0.86 (–1.34 to –0.38)^j^	–2.95 (–4.41 to –1.49)^j^	–0.02 (–0.03 to –0.004)^o,m^	–2.42 (–3.25 to –1.61)^j^	–3.98 (–6.96 to –0.99)^o^	–0.13 (–1.91 to 1.65)	2.80 (1.89 to 3.72)^j^
	At least MVPA+sleep	–0.97 (–1.17 to –0.76)^j^	–3.06 (–3.68 to –2.43)^j^	–0.01 (–0.02 to –0.01)^j^	–1.82 (–2.17 to –1.47)^j^	–1.12 (–2.40 to –0.17)	–0.22 (–0.99 to 0.54)	2.76 (2.37 to 3.14)^j^
	At least SB+sleep	–0.89 (–1.54 to –0.24)^o^	–2.75 (–4.72 to –0.79)^o^	–0.02 (–0.03 to –0.002)^l^	–2.53 (–3.63 to –1.43)^j^	–3.98 (–8.00 to 0.03)	0.33 (–2.06 to 2.73)	3.49 (2.27 to 4.72)^j^
**Number of guidelines met^p^**								
	Meeting 1	–0.93 (–1.14 to –0.71)^j^	–5.48 (–7.47 to –3.48)^j^	–0.02 (–0.02 to –0.01)^j^	–1.50 (–1.86 to –1.13)^j^	0.86 (–0.50 to 2.21)	0.02 (–0.79 to 0.83)	2.26 (1.86 to 2.66)^j^
	Meeting 2	–1.64 (–1.89 to –1.38)^j^	–4.97 (–5.75 to –4.20)^j^	–0.02 (–0.03 to –0.02)^j^	–2.91 (–3.34 to –2.47)^j^	–0.55 (–2.16 to 1.06)	–0.29 (–1.25 to 0.67)	4.30 (3.82 to 4.77)^j^
	Meeting 3	–1.84 (–2.50 to –1.18)^j^	–2.56 (–3.21 to –1.91)^j^	–0.03 (–0.05 to –0.02)^j^	–4.13 (–5.25 to –3.01)^j^	–3.64 (–7.79 to 0.51)	0.28 (–2.20 to 2.75)	5.90 (4.67 to 7.13)^j^
	Trend analysis	–0.77 (–0.89 to –0.65)^j^	–2.35 (–2.72 to –1.99)^j^	–0.02 (–0.02 to –0.01)^j^	–1.44 (–1.64 to –1.23)^j^	–0.51 (–1.27 to 0.24)	–0.11 (–0.56 to 0.34)	2.11 (1.89 to 2.33)^j^

^a^All models were adjusted for age, gender, residence, education, marital status, chronic diseases, smoking, alcohol consumption, and municipality economic status.

^b^Independent variables were meeting movement guidelines and dependent variables were health outcomes.

^c^WC: waist circumference.

^d^WHR: waist-hip ratio.

^e^PBF: percentage body fat.

^f^SBP: systolic blood pressure.

^g^DBP: diastolic blood pressure.

^h^Not meeting individual guidelines as the reference group.

^i^MVPA: moderate-to-vigorous physical activity.

^j^*P*<.001.

^k^SB: sedentary behavior.

^l^*P*<.05.

^m^The analysis was not robust in terms of the sensitivity analysis, with the exclusion of participants who were obese and those who had moderate or severe depressive symptoms.

^n^Not meeting specific guideline combinations as the reference group.

^o^*P*<.01.

^p^Not meeting any guideline as the reference group.

**Table 4:** Associations of adherence to 24-hour movement guidelines with mental health outcomes (N=4562).

Meeting movement guidelines^a,b^		Depression, *B* (95% CI)		Loneliness, *B* (95% CI)
**Meeting individual guidelines^c^**				
	At least MVPA^d^	–1.99 (–2.18 to –1.80)^e^	–3.57 (–4.04 to –3.09)^e^	
	At least SB^f^	–2.05 (–2.55 to –1.54)^e^	–1.95 (–3.19 to –0.71)^g,h^	
	At least sleep	–2.60 (–2.79 to –2.42)^e^	–4.00 (–4.47 to –3.53)^e^	
**Meeting specific guideline combinations^i^**				
	At least MVPA+SB	–2.04 (–2.54 to –1.53)^e^	–1.89 (–3.14 to –0.64)^j,h^	
	At least MVPA+sleep	–2.01 (–2.23 to –1.80)^e^	–3.53 (–4.05 to –3.00)^e^	
	At least SB+sleep	–1.76 (–2.45 to –1.08)^e^	–1.14 (–2.82 to 0.54)	
**Number of guidelines met^k^**				
	Meeting 1	–3.28 (–3.48 to –3.07)^e^	–5.11 (–5.65 to –4.58)^e^	
	Meeting 2	–4.43 (–4.67 to –4.19)^e^	–7.35 (–7.99 to –6.72)^e^	
	Meeting 3	–4.75 (–5.37 to –4.14)^e^	–5.95 (–7.59 to –4.32)^e^	
	Trend analysis	–2.05 (–2.16 to –1.93)^e^	–3.28 (–3.58 to –2.98)^e^	

^a^All models were adjusted for age, gender, residence, education, marital status, chronic diseases, smoking, alcohol consumption, and municipality economic status.

^b^Independent variables were meeting movement guidelines and dependent variables were health outcomes.

^c^Not meeting individual guidelines as the reference group.

^d^MVPA: moderate-to-vigorous physical activity.

^e^*P*<.001.

^f^SB: sedentary behavior.

^g^*P*<.05.

^h^The analysis was not robust in terms of the sensitivity analysis, with the exclusion of participants who were obese and those who had moderate or severe depressive symptoms.

^i^Not meeting specific guideline combinations as the reference group.

^j^*P*<.01.

^k^Not meeting any guideline as the reference group.

### Sensitivity Analyses

The outcomes of the sensitivity analyses, which excluded participants with obesity and those with moderate to severe depressive symptoms, aligned with the primary findings, except for 7 discrepancies (Multimedia Appendix 1). In particular, the association between adherence to SB guidelines and loneliness (*P*=.06) was not significant in the sensitivity analysis. Similarly, adhering to sleep guidelines was not significantly associated with BMI (*P*=.49), waist circumference (*P*=.78), or PBF (*P*=.42). Furthermore, the correlation between adherence to MVPA+SB guidelines and WHR (*P*=.10) and loneliness (*P*=.07) was not statistically significant among participants, excluding those with obesity and those with moderate to severe depressive symptoms. The e-values showed that the main findings of the association examination are unlikely to be nullified by unmeasured confounders (all e-values were >1) (Multimedia Appendix 1).

## Discussion

### Principal Findings

This study provides timely evidence on the prevalence and correlates of adherence to 24-hour movement guidelines as well as its associations with physical and mental health outcomes among older adults. The results showed that only 1.8% (83/4562) of participants met all 3 movement guidelines, while 32.1% (1466/4562), 3.4% (155/4562), and 66.4% (3031/4562) met individual behavioral guidelines for MVPA, SB, and sleep, respectively (aim 1). Participants who were older, were female, and lived in municipalities with a lower economic status were less likely to comply with all 3 movement guidelines, while those who lived in urban areas, had higher education levels, and had no chronic diseases showed a higher adherence to specific individual movement guidelines or combinations of movement guidelines (aim 2). With regard to aim 3, adherence to either individual movement guidelines or combinations of movement guidelines was associated with a higher level of physical fitness and lower levels of BMI, waist circumference, WHR, PBF, depressive symptoms, and loneliness. This did not extend to the relationship between adherence to SB+sleep guidelines and loneliness. Furthermore, adherence to movement guidelines did not correlate with 2 blood pressure indicators, except when adhering to SB or MVPA+SB guidelines.

Regarding guideline adherence (aim 1), the percentage of Chinese older adults meeting all 3 movement guidelines was lower than that of other age groups as reported in previous national surveys (eg, Chinese children and adolescents: 2.1%; Chinese caregivers of preschoolers: 15.1%; Canadian adults: 7.1%; and Thailand adults: 21.3%) [36,38,41,42]. This is consistent with previous studies, which indicated a poorer adherence to 24-hour movement guidelines among older adults compared with other age groups [30,37,38]. Age-related decreases in PA and sleep and increases in sedentary time have been demonstrated in previous studies [61,62]. This is not surprising as older adults generally experience physical and cognitive hypofunction with age, coupled with worries of life transition, diseases, and death [63], which, to some extent, may weaken the antecedents of behavioral initiation (eg, perceived capability and motivation), eventually leading to unhealthy patterns of movement behaviors (eg, physical inactivity, prolonged SB, and insufficient sleep) [64]. In addition to this explanation, the time frame of data collection should be considered. The data in our study were gathered during the COVID-19 period, and at that time, local preventive measures were still being undertaken (eg, mandatory quarantine, physical distancing, and emergent closure of some public areas). This might have contributed to the low adherence rate of meeting 24-hour movement guidelines among Chinese older adults [65,66]. Overall, the above findings underline the long-term requirement of effective behavioral promotion strategies and policy making. In addition, as there is a lack of evidence on the adherence of older adults to 24-hour movement guidelines, we could not make a comparison with previous studies using the same age group, which implies that more surveillance studies on this topic are warranted.

Regarding the correlates of movement guideline adherence among Chinese older adults (aim 2), we found that participants who were female and who were older were less likely to comply with movement guidelines, which is in accordance with the findings of previous studies involving children and adults [35-38]. Interestingly, we also found a lower adherence to all 3 movement guidelines among participants who lived in municipalities ranking <9th in terms of GDP. This might reflect the fact that compared with those who lived in cities with a lower economic status and lower modernization, Chinese older adults who lived in cities with a higher GDP had a healthy lifestyle. For other covariates, some were only associated with specific guideline adherence (eg, a higher adherence to MVPA and sleep was found among participants living in urban areas, yet residence was not associated with SB guideline adherence), where mixed results were also demonstrated in previous studies with children and adults [30,35,42,67]. Overall, the above findings imply that future health promotion programs and policy making should take age, gender, and municipality economic status differences into account. For example, more effective strategies for motivating female individuals and older age groups are needed (eg, designing programs based on participants’ preferences, tailoring the intervention content based on preidentified psychosocial determinants targeting these samples, and introducing a more supportive policy for these economically disadvantaged municipalities). In addition, more research on examining the role of other demographic factors is warranted.

Regarding the association between movement guideline adherence and health-related outcomes among Chinese older adults (aim 3), we found that adherence to movement guidelines, individually or in combination, was associated with greater physical health indicators, including BMI, PBF, waist circumference, WHR, and physical fitness. These findings are consistent with previous findings from children and adult populations [37-42], suggesting the broader applicability of these guidelines. Notably, our research showed that only adherence to SB or MVPA+SB guidelines was associated with a lower systolic pressure, contrasting with the lack of an association between adherence to other guidelines and blood pressure indicators. The limited evidence on the relationship between 24-hour movement guideline adherence and the health of older adults constrains a comprehensive comparison with existing studies. Notably, current research on movement behaviors and their relationships with blood pressure indicators (eg, using a compositional data analysis) has presented mixed findings among older adults [27,68,69]. This discrepancy may be attributed to different types of PAs (eg, muscle strength training and aerobic exercise) and dietary factors (eg, sodium intake) [70,71]. In relation to blood pressure among older adults, the quality of PA (eg, specific modality) and dietary patterns may serve as more sensitive and significant correlates than the quantity of PA (eg, minutes per week). Moreover, age-related physiological changes may reduce the sensitivity of blood pressure to movement behaviors [72], and the prevalent use of antihypertensive medications within the elderly population could also obscure the potential benefits of adhering to movement guidelines (eg, achieving the recommended levels of MVPA) [73]. Nevertheless, these assumptions were not explored in our study, highlighting the need for systematic investigations in future research.

For mental health outcomes, we found that adherence to movement guidelines was associated with lower levels of depressive symptoms and loneliness, except adherence to SB+sleep guidelines, which showed no significant relationship with loneliness. Engaging in a variety of physical activities, especially peer-based or group-oriented ones, significantly benefits the emotional and social well-being of elderly people [74]. The influence of SB and sleep duration on loneliness among older adults has not been convincingly demonstrated by prior research [27,75,76]. It is important to recognize that not all sedentary activities exert the same effect on mental well-being. Engaging in socially interactive sedentary activities (eg, internet-based social activities and playing chess with friends) may actually contribute to reducing depressive symptoms and feelings of loneliness [76,77]. The findings of our study support the connection between adherence to movement guidelines and mental health outcomes among older adults. However, evaluating activity solely by its duration does not provide a comprehensive understanding of its effect on mental health. Future research is needed to investigate the specific mechanisms by which movement behaviors contribute to the improvement of mental health, thereby informing the development of more effective health promotion initiatives.

Finally, the results of the sensitivity analyses corroborate the robustness of our data analyses, with certain exceptions noted in the associations between adherence to SB guidelines and loneliness; adherence to sleep guidelines and BMI, waist circumference, and PBF; adherence to MVPA+SB guidelines and WHR; and adherence to MVPA+sleep guidelines and loneliness. These exceptions suggest that the weight status and the severity of depressive symptoms might affect the solidity of our findings, warranting further investigations in future studies. Additionally, our e-value analyses consistently yielded values exceeding 1, affirming the resilience of our findings against the influence of unmeasured confounders and therefore strengthening the stability of our results. These findings validate the integrity of the associations identified in our study. It is worth noting that our observations also reveal that lower adherence to movement guidelines corresponds with reduced e-values, suggesting a comparatively increased susceptibility to unobserved confounders. This discrepancy indicates the importance of cautious interpretation and necessitates further exploration of potential unmeasured confounders, such as social and environmental factors, to enhance our comprehension of the intricate relationship between adherence to 24-hour movement guidelines and health-related outcomes.

### Limitations

Several limitations should be noted. First, although our study applied stratified random sampling with a large sample size, the study findings to some extent could only reflect the behavioral profiles of Chinese older adults living in the central region of China, and the generalizability to other regions (eg, the north and south of China) and different cultural contexts should be further examined. Second, the causal relationship between movement behaviors and health outcomes could not be well supported by the cross-sectional design. A further examination using longitudinal and experimental designs is warranted. Moreover, all the movement behaviors were evaluated by self-reported items. Although these kinds of measures have been well validated and have been shown to have advantages in several aspects (eg, could reach wider participants and be more feasible in a large sample surveillance), they might lead to measurement biases (eg, recall bias and social disabilities). Objective measures for movement behaviors are warranted in future research. In addition, for the correlates of movement guideline adherence, our findings could only explain a small percentage of the variance. Further examination of the potential correlates (eg, psychosocial and environmental factors) should be performed in future studies.

### Conclusions

This study found that only 1.8% of Chinese older adults adhered to 24-hour movement guidelines. Older age, female sex, and lower municipality economic levels were associated with poor adherence to movement guidelines among Chinese older adults. Importantly, adherence to either individual or combined movement guidelines was associated with better physical and mental health outcomes. These findings suggest the potential benefits of promoting a holistic lifestyle encompassing adequate MVPA, reduced SB, and sufficient sleep for improving the physical and mental well-being of elderly populations.

## Appendix

Multimedia Appendix 1 Results of sensitivity analyses.

## Abbreviations

DBP: diastolic blood pressure
GDP: gross domestic product
HSHC: health surveillance of Hubei China
MVPA: moderate-to-vigorous physical activity
OR: odds ratio
PA: physical activity
PAR-Q: Physical Activity Readiness Questionnaire
PBF: percentage body fat
PHQ-9: Patient Health Questionnaire-9
SB: sedentary behavior
SBP: systolic blood pressure
WHR: waist-hip ratio
