# Supplementary material for: Adherence to 24-Hour Movement Guidelines Among Chinese Older Adults: Prevalence, Correlates, and Associations With Physical and Mental Health Outcomes
Source: JMIR Public Health Surveill. 2024 Jun 13;10:e46072. doi: 10.2196/46072 (PMC11211711; doi:10.2196/46072)
Supplement: Multimedia Appendix 1 [file publichealth_v10i1e46072_app1.pdf]

Multimedia Appendix 1. Results of sensitivity analyses.

Table S1. Sensitivity analyses for the correlates of meeting 24-hour movement guidelines in the study sample (n=3,870)

**Variable**	**MVPA**	**SB**	**Sleep**	**MVPA+SB**	**MVPA+Sleep**	**SB+Sleep**	**MVPA+SB+Sleep**
	OR **(95%CI)**	***OR*** **(95%CI)**	***OR*** **(95%CI)**	***OR*** **(95%CI)**	***OR*** **(95%CI)**	***OR*** **(95%CI)**	***OR*** **(95%CI)**
Age	0.98(0.96,0.99) **	0.94(0.90,0.97) **	0.99(0.98,1.00)	0.94(0.90,0.97) **	0.98(0.97,1.00)	0.94(0.90,0.99) *	0.94(0.90,0.99) *
Gender							
Male (Ref.)	N/A	N/A	N/A	N/A	N/A	N/A	N/A
Female	0.57(0.49,0.67) ***	0.41(0.27,0.62) ***	0.66(0.56,0.79) ***	0.40(0.26,0.60) ***	0.59(0.50,0.71) ***	0.32(0.18,0.56) ***	0.32(0.18,0.56) ***
Residence							
Urban (Ref.)	N/A	N/A	N/A	N/A	N/A	N/A	N/A
Countryside	0.77(0.67,0.88) ***	0.80(0.56,1.14)	0.79(0.68,0.91) **	0.77(0.54,1.10)	0.79(0.68,0.92) **	0.79(0.49,1.27)	0.79(0.49,1.27)
Education							
Primary school and below (Ref.)	N/A	N/A	N/A	N/A	N/A	N/A	N/A
Secondary school	1.19(1.02,1.39) *	0.95(0.65,1.38)	1.11(0.95,1.30)	0.98(0.66,1.43)	1.19(1.01,1.41) *	1.40(0.80,2.45)	1.40(0.80,2.45)
College and above	1.34(1.06,1.70) *	0.76(0.43,1.35)	1.90(1.44,2.50) ***	0.78(0.44,1.38)	1.44(1.12,1.84) **	1.29(0.61,2.73)	1.29(0.61,2.73)
Marital status							
Married (Ref.)	N/A	N/A	N/A	N/A	N/A	N/A	N/A
Single/divorced/widowed	1.16(0.95,1.42)	0.98(0.56,1.71)	1.00(0.81,1.24)	0.92(0.52,1.64)	1.20(0.96,1.50)	0.77(0.33,1.82)	0.77(0.33,1.82)
Chronic disease							
No (Ref.)	N/A	N/A	N/A	N/A	N/A	N/A	N/A
Yes	0.95(0.83,1.09)	1.29(0.92,1.82)	0.71(0.61,0.82) ***	1.29(0.92,1.82)	0.94(0.81,1.10)	1.12(0.71,1.78)	1.12(0.71,1.78)
Smoking							
Not currently (Ref.)	N/A	N/A	N/A	N/A	N/A	N/A	N/A
Yes, but not everyday	0.58(0.39,0.84) **	0.50(0.19,1.32)	1.05(0.70,1.58)	0.50(0.19,1.32)	0.71(0.47,1.08)	0.41(0.09,1.79)	0.41(0.09,1.79)
Yes, almost everyday	1.12(0.91,1.39)	0.92(0.58,1.44)	1.08(0.85,1.36)	0.90(0.57,1.42)	1.21(0.96,1.52)	0.93(0.51,1.73)	0.93(0.51,1.73)
Alcohol							
Never (Ref.)	N/A	N/A	N/A	N/A	N/A	N/A	N/A
Seldomly	1.43(1.16,1.78) **	1.14(0.69,1.86)	1.11(0.87,1.41)	1.15(0.70,1.88)	1.36(1.08,1.70) **	0.95(0.49,1.85)	0.95(0.49,1.85)
Often	1.24(0.99,1.57)	1.44(0.89,2.34)	0.78(0.61,1.00) *	1.45(0.89,2.35)	1.06(0.83,1.36)	1.04(0.53,2.03)	1.04(0.53,2.03)
Municipality economic status							
< 9th of GDP (Ref.)	N/A	N/A	N/A	N/A	N/A	N/A	N/A
⩾ 9th of GDP	0.93(0.81,1.07)	1.46(1.03,2.07) *	0.86(0.74,0.99) *	1.46(1.03,2.07) *	0.95(0.81,1.10)	1.61(1.00,2.59)	1.61(1.00,2.59)
R^2	0.05	0.05	0.04	0.05	0.04	0.06	0.06

Note. Ref. = reference group; MVPA = moderate to vigorous physical activity; SB = sedentary behavior; 95%CI = 95% confidence interval; N/A = not applicable; $^{*}P$<0.05, $^{**}P$<0.01, $^{***}P$<0.001.

Table S2. Sensitivity analyses for the associations of meeting 24-hour movement guidelines with physical health outcomes (n=3,870).

**Meeting movement guidelines**[1]	**BMI (kg/m^2)**	**Waist circumference (cm)**	**WHR**	**PBF (%)**	**SP (mmHg)**	**DP (mmHg)**	**Physical fitness**
	***B* (95%CI)**	***B* (95%CI)**	***B* (95%CI)**	***B* (95%CI)**	***B* (95%CI)**	***B* (95%CI)**	***B* (95%CI)**
Meeting individual guideline[2]							
At least MVPA	0.77 (-0.92, -0.61)***	-2.93 (-3.47, -2.39)***	-0.01 (-0.02, -0.01)***	-1.94 (-2.25, -1.63)***	-1.17 (-2.41, 0.07)	-0.07 (-0.81, 0.67)	2.41 (2.06, 2.76)***
At least SB	-0.49 (-0.88, -010)*	-2.02 (-3.36, -0.67)**	-0.01 (-0.02, -0.001)*	-1.97 (-2.75, -1.20)***	-3.56 (-6.61, -0.50)*	-0.01 (-1.83, 1.80)	2.35 (1.47, 3.23)***
At least sleep	**0.06 (-0.11, 0.23)**	**-0.08 (-0.66, 0.49)**	-0.01 (-0.01, -0.001)*	**0.14 (-0.20, 0.47)**	1.22 (-0.08, 2.52)	0.54 (-0.23, 1.32)	0.58 (0.20, 0.95)**
Meeting specific guidelines combinations[3]							
At least MVPA+SB	-0.51 (-0.90, -0.11)*	-2.19 (-3.54, -0.83)**	**-0.01 (-0.02, 0.002)**	-2.04 (-2.82, -1.26)***	-3.96 (-7.03, -0.88)*	-0.16 (-1.99, 1.68)	2.35 (1.47, 3.24)***
At least MVPA+Sleep	-0.49 (-0.66, -0.32)***	-2.10 (-2.69, -1.51)***	-0.01 (-0.01, -0.004)***	-1.26 (-1.60, -0.92)***	-0.84 (-2.19, 0.51)	0.02 (-0.78, 0.82)	2.21 (1.83, 2.59)***
At least SB+Sleep	-0.58 (-1.11, -0.05)*	-1.96 (-3.78, -0.13)*	-0.01 (-0.03, 0.004)	-2.19 (-3.24, -1.13)***	-4.14 (-8.28, 0.006)	0.34 (-2.12, 2.80)	3.09 (1.90, 4.28)***
The number of guidelines met[4]							
Meeting one	-0.30 (-0.50, -0.10) **	-1.16 (-1.85, -0.47) ***	-0.01 (-0.02, -0.01) ***	-0.73 (-1.12, -0.33) ***	1.52 (-0.05, 3.10)	0.71 (-0.23, 1.65)	0.94 (0.50,1.39) ***
Meeting two	-0.71 (-0.94, -0.48) ***	-3.07 (-3.86, -2.28) ***	-0.02 (-0.02, -0.01) ***	-.1.82 (-2.26, -1.36) ***	0.28 (-1.52, 2.08)	0.46 (-0.61, 1.53)	2.84 (2.33, 3.35) ***
Meeting all	-0.96 (-1.51, -.0.40) ***	-3.48 (-5.38, -1.58) ***	-0.02 (-0.04, -0.01) **	-3.11 (-4.21, -2.02) ***	-3.19 (-7.52, 1.14)	0.88 (-1.70, 3.45)	4.45 (3.22, 5.67) ***
Trend analysis	-0.35 (-0.46, -0.25) ***	-1.49 (-1.86, -1.13) ***	-0.01 (-0.01, -0.005) ***	-0.96 (-1.17, -0.75) ***	-0.29 (-1.12, 0.54)	-0.19 (-0.30, 0.68)	1.49 (1.25, 1.73) ***

Note. [1]Independent variables were meeting movement guidelines and dependent variables were health outcomes; [2]Not meeting individual guideline as reference group; [3]Not meeting specific guideline combinations as reference group; [4]Not meeting any guideline as reference group; MVPA= moderate-to-vigorous physical activity; SB=sedentary behavior; All models were adjusted for age, gender, residence, education, marital status, chronic disease, smoking, alcohol, and municipality economic status; BMI = body mass index; WHR = waist-hip ratio; PBF = percentage body fat; SBP = systolic pressure; DBP = diastolic pressure; 95%CI = 95% confidence interval; $^{***}P < 0.001$, $^{**}P < 0.01$, $^{*}P < 0.05$.

Table S3. Sensitivity analyses for the associations of meeting 24-hour movement guidelines with mental health outcomes (n = 3,870).

**Meeting movement guidelines**[1]	**Depression**	**Loneliness**
	B **(95%CI)**	***B*** **(95%CI)**
Meeting individual guideline[2]		
At least MVPA	-1.35 (-1.51, -1.19)***	-2.38 (-2.84, -1.92)***
At least SB	-1.60 (-2.00, -1.20)***	**-1.15 (-2.30, 0.01)**
At least sleep	-1.55 (-1.72, -1.39)***	-2.09 (-2.57, -1.60)***
Meeting specific guidelines combinations[3]		
At least MVPA+SB	-1.59 (-1.99, -1.18)***	**-1.08 (-2.25, 0.08)**
At least MVPA+Sleep	-1.44 (-1.61, -1.27)***	-2.48 (-2.98, -1.97)***
At least SB+Sleep	-1.34 (-1.89, -0.79)***	-0.43 (-1.99, 1.14)
The number of guidelines met[4]		
Meeting one	-1.95 (-2.14, -1.76) ***	-2.64 (-3.22, -2.06) ***
Meeting two	-2.99 (-3.21, -2.77) ***	-4.62 (-5.29, -3.96) ***
Meeting all	-3.31 (-3.83, -2.78) ***	-3.28 (-4.88, -1.68) ***
Trend analysis	-1.35 (-1.46, -1.25) ***	-2.00 (-2.31, -1.69)***

Note. [1]Independent variables were meeting movement guidelines and dependent variables were health outcomes; [2]Not meeting individual guideline as reference group; [3]Not meeting specific guideline combinations as reference group; [4]Not meeting any guideline as reference group; MVPA= moderate-to-vigorous physical activity; SB=sedentary behavior; All models were adjusted for age, gender, residence, education, marital status, chronic disease, smoking, alcohol, and municipality economic status; All models were adjusted for age, gender, residence, education, marital status, chronic disease, smoking, alcohol, and municipality economic status; 95%CI = 95% confidence interval; $^{***}P < 0.001$, $^{**}P < 0.01$.

Table S4 E-values for the associations of meeting 24-hour movement guidelines with physical health outcomes (n=4,562)

**Meeting movement guidelines**[a]	**BMI (kg/m^2)**	**WC (cm)**	**WHR**	**PBF (%)**	**SP (mmHg)**	**DP (mmHg)**	**Physical fitness**
	**E-values (CI)**	**E-values (CI)**	**E-values (CI)**	**E-values (CI)**	**E-values (CI)**	**E-values (CI)**	**E-values (CI)**
Meeting individual guideline[b]							
At least MVPA	2.23 (2.07)	2.26 (2.09)	1.92 (1.77)	2.11 (1.98)	1.29 (1.00)	1.15 (1.00)	1.82 (1.75)
At least SB	1.89 (1.48)	1.95 (1.54)	1.54 (1.00)	2.08 (1.76)	1.67 (1.21)	1.02 (1.00)	1.78 (1.58)
At least sleep	1.60 (1.43)	1.55 (1.39)	1.50 (1.34)	1.41 (1.27)	1.15 (1.00)	1.08 (1.00)	1.52 (1.44)
Meeting specific guidelines combinations[c]							
At least MVPA+SB	1.91 (1.48)	2.00 (1.58)	1.92 (1.47)	2.10 (1.78)	1.73 (1.28)	1.12 (1.00)	1.78 (1.58)
At least MVPA+Sleep	2.01 (1.83)	2.03 (1.85)	1.54 (1.38)	1.86 (1.73)	1.30 (1.00)	1.16 (1.00)	1.77 (1.69)
At least SB+Sleep	1.93 (1.36)	1.94 (1.37)	1.92 (1.31)	2.14 (1.71)	1.73 (1.00)	1.20 (1.00)	1.94 (1.66)
The number of guidelines met[d]							
Meeting one	1.97 (1.78)	1.88 (1.7)	1.76 (1.58)	1.74 (1.6)	1.25 (1)	1.05 (1)	2.18 (1.99)
Meeting two	2.64 (2.39)	2.62 (2.37)	2.07 (1.85)	2.3 (2.12)	1.2 (1)	1.87 (1.63)	3.26 (2.98)
Meeting three	2.85 (2.2)	2.79 (2.15)	2.36 (1.76)	2.85 (2.34)	1.68 (1)	1.18 (1)	4.36 (3.5)
Trend analysis	1.83 (1.73)	1.82 (1.72)	1.61 (1.54)	1.71 (1.64)	1.19 (1.16)	1.11 (1.11)	1.63 (1.58)

Note. reference group = meeting none of movement guidelines; BMI = body mass index; WHR = waist-hip ratio; PBF = percentage body fat; SBP = systolic pressure; DBP = diastolic pressure; CI = confidence interval.

Table S5 E-values for the associations of meeting 24-hour movement guidelines with mental health outcomes (n=4,562)

**Meeting movement guidelines**[a]	**Depression**	**Loneliness**
	**E-values (CI)**	**E-values (CI)**
Meeting individual guideline[b]		
At least MVPA	2.83 (2.64)	2.38 (2.21)
At least SB	2.89 (2.41)	1.81 (1.39)
At least sleep	3.47 (3.27)	2.55 (2.37)
Meeting specific guidelines combinations[c]		
At least MVPA+SB	2.88 (2.40)	1.79 (1.36)
At least MVPA+Sleep	2.85 (2.64)	2.37 (2.18)
At least SB+Sleep	2.61 (2.01)	1.54 (1.00)
The number of guidelines met[d]		
Meeting one	4.3 (4.04)	3 (2.78)
Meeting two	6.12 (5.7)	4.09 (3.76)
Meeting three	6.73 (5.61)	3.38 (2.67)
Trend analysis	2.88 (2.77)	2.27 (2.21)

Note. reference group = meeting none of movement guidelines; BMI = body mass index; WHR = waist-hip ratio; PBF = percentage body fat; SBP = systolic pressure; DBP = diastolic pressure; CI = confidence interval.
